# Characterization of the complete mitochondrial genome of *Tylorida striata* (Araneae: Tetragnathidae) and phylogenetic analysis

**DOI:** 10.1080/23802359.2019.1698367

**Published:** 2019-12-13

**Authors:** Kang-Kang Xu, Shu-Yan Yan, Can Li, Da-Xing Yang

**Affiliations:** Guizhou Provincial Key Laboratory for Rare Animal and Economic Insect of the Mountainous Region, College of Biology and Environmental Engineering, Guiyang University, Guiyang, Guizhou, China

**Keywords:** *Tylorida striata*, Tetragnathidae, Mitogenome

## Abstract

The complete mitochondrial genome of *Tylorida striata* (GenBank accession number MN615900) is 14,422 bp in length with a high AT bias of 71.73%. It contains 13 protein-coding genes (PCGs), 22 transfer RNA genes (tRNAs), 2 ribosomal RNA (rRNA) genes, and a putative control region. The gene order of *T. striata* was identical to other spider mitogenomes. Eleven tRNAs (*trnW*, *trnY*, *trnC*, *trnD*, *trnG*, *trnL_2_*, *trnR*, *trnF*, *trnP*, *trnI*, and *trnL_1_*) lose the TΨC-stems, whereas two tRNAs (*trnS_1_* and *trnS_2_*) lack the dihydrouracil (DHU) arm. Phylogenetic tree based on concatenated amino acid sequences of 13 PCGs showed that *T. striata* is clustered with two Tetragnathidae species and recovered as sister of other two Tetragnathids.

The big-jawed spider *Tylorida striata* belongs to the family of Tetragnathidae, which includes about 9000 described species in 50 genera (Kulkarni and Yadav 2015; Platnick 2015). Most of these spiders are important predators of several insects and usually live in grassy places and swamps (Zhang et al. 2002). In this study, adult species of *T*. *striata* were collected from Maolan Nature Reserve in Libo County, Guizhou Province, China (N25°16′, E107°58′), and preserved in spider specimen room of Guiyang University with an accession number GYU-GZML-23.

The complete mitogenome sequence of *T*. *striata* is deposited in GenBank under accession number MN615900. It is a circular molecule of 14,422 bp in length and contains sets of genes, including 13 protein-coding genes (PCGs), 22 transfer RNA genes (tRNAs), 2 ribosomal RNA genes (*12S rRNA* and *16S rRNA*), and a putative control region (Boore 1999; Yan et al. 2019). The gene order of *T. striata* was identical with other spider mitogenomes (Li et al. 2016; Yang et al. 2019). Fourteen genes were transcribed on the minor strand (N-strand), while the others were oriented on the major strand (J-strand). The overall base composition of *T. striata* mitogenome is A (31.27%), C (10.19%), G (18.08%), and T (40.46%), with a high AT bias of 71.73%. The AT-skew and GC-skew of this genome were −0.128 and 0.279, respectively.

There were 5 intergenic spacer regions comprising a total length of 23 bp and the largest spacer (8 bp) was located between *nad1* and *trnL_1_*. Gene overlaps were found at 23 gene junctions, the longest 35 bp overlapping located between *trnE* and *trnF*. The length of 22 tRNAs varied between 48 bp (*trnC*) and 77 bp (*trnM*), with a mean length of 59 bp. Thirteen tRNAs could not be folded into typical cloverleaf-shaped secondary structures. Among them, eleven tRNAs (*trnW*, *trnY*, *trnC*, *trnD*, *trnG*, *trnL_2_*, *trnR*, *trnF*, *trnP*, *trnI*, and *trnL_1_*) lost the TΨC-stem and two tRNAs (*trnS_1_* and *trnS_2_*) lacked the dihydrouracil (DHU) arm. The *16S rRNA* (1014 bp) is located between *trnL_1_* and *trnV*, and *12S rRNA* (691 bp) reside between *trnV* and *trnQ*, and their A + T contents were 76.23 and 78.00%, respectively. The control region is located between *trnQ* and *trnM* with a length of 895 bp long and the A + T content is 75.87%. Ten PCGs initiate with typical ATN start codons (ATT for *atp8*, *nad1*, *nad2*, *nad4L*, *nad5*, and *nad6*; ATA for *nad4* and *cob*; ATG for *atp6* and *nad3*), two PCGs (*cox2* and *cox3*) use TTG and *cox1* is initiated with TTA. Ten PCGs use the typical termination codons TAA and TAG in *T*. *striata*, while only three PCGs (*nad4L*, *nad5*, and *cob*) stop with the incomplete termination single T.

Based on the concatenated amino acid sequences of 13 PCGs from *T*. *striata* and 14 other spiders, the neighbor-joining method was used to reveal the phylogenetic relationship among these spiders. The result showed that *T. striata* is clustered with two Tetragnathidae species (Figure 1). Within the Tetragnathidae clade, *T. striata* is recovered as sister to the other two Tetragnathids.

**Figure 1.**
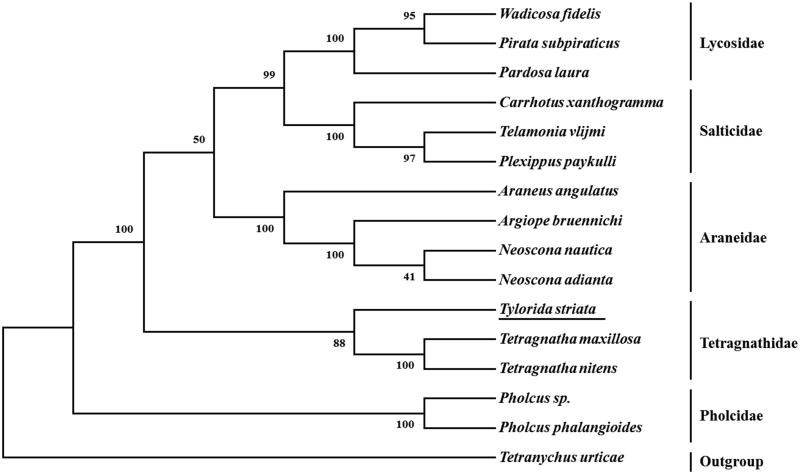
Phylogenetic tree showing the relationship between *Tylorida striata* and 14 other representative spiders based on neighbor-joining method. GenBank accession numbers used in the study are the following: *Araneus angulatus* (KU365988), *Argiope bruennichi* (NC_024281), *Carrhotus xanthogramma* (NC_005942), *Neoscona adianta* (KR259805), *Neoscona nautica* (KR259804), *Pardosa laura* (KM272948), *Pholcus phalangioides* (NC_020324), *Pholcus sp.* (KJ782458), *Pirata subpiraticus* (NC_025523), *Plexippus paykulli* (NC_024877), *Telamonia vlijmi* (NC_024287), *Tetragnatha maxillosa* (KP306789), *Tetragnatha nitens* (KP306790), *Tetrancychus urticae* (EU345430), *Wadicosa fidelis* (NC_026123). *Tetrancychus urticae* was used as an outgroup. The spider determined in this study is underlined.
